# Evolution of Microstructures and Mechanical Properties of Nb-V Alloyed Ultra-High Strength Hot Stamping Steel in Austenitizing Process

**DOI:** 10.3390/ma15228197

**Published:** 2022-11-18

**Authors:** Shuang Liu, Songyuan Ai, Mujun Long, Yi Feng, Jingjun Zhao, Yan Zhao, Xiang Gao, Dengfu Chen, Mingtu Ma

**Affiliations:** 1 Laboratory of Metallurgy and Materials, College of Materials Science and Engineering, Chongqing University, Chongqing 400044, China; 2 China Automotive Engineering Research Institute Co., Ltd., Chongqing 401122, China; 3 Beijing Institute of Technology Chongqing Innovation Center, Chongqing 400000, China

**Keywords:** hot stamping steel, mechanical properties, Nb-V alloyed, microstructures

## Abstract

Clarifying the influence of Nb and V microalloying on the ultra-high strength hot stamping steel (UHSHSS) and exploring appropriate process parameters are the basis for effectively regulating properties of the final product. In this study, the effects of different austenitizing temperatures and holding times on the phase transitions, grain sizes and mechanical properties of 22MnB5NbV with Nb and V alloyed are studied by using JMatPro thermodynamic calculations and experiments. By comparing with 22MnB5 without Nb and V alloyed, the effects of Nb and V elements on quenching microstructures, grain sizes and mechanical properties of UHSHSS are analyzed. The suitable austenitizing process parameters of 22MnB5NbV have been obtained. The results show that the grain size of Nb-V-alloyed UHSHSS grows with the increase in the austenitizing temperature and holding time. The 22MnB5NbV steel can be completely austenitized while the austenitizing temperatures ≥870 °C and holding time ≥3 min. Combined with the actual production process, the best austenitizing temperature and holding time are 930 °C and 3 min. Under these conditions, the average grain size is 7.7 μm, the tensile strength, elongation and strength-ductility product are 1570.8 MPa, 6.6% and 10.4 GPa·%, respectively. With the addition of Nb and V elements, the nanoscale precipitates lead to the refinement of the quenched structure and grain size, and the comprehensive properties of UHSHSS have been effectively promoted, in which the elongation and strong-plastic products are increased by ~0.6% and ~1.2 GPa·%, respectively.

## 1. Introduction

With the extensive use of lightweight technology in the automotive field, hot stamping steel with high strength has been widely used in the production of special automotive parts, due to its excellent mechanical properties [1,2,3,4]. The 22MnB5 steel with a tensile strength of about 1500 MPa is one of the typical hot stamping steels for automobiles, in which the microstructure is high-strength martensite at room temperature. Unfortunately, the lower elongation and poor ductility of 22MnB5 steel limits its further development in the automotive field [5,6,7]. Obviously, the production process and composition control together determine the final mechanical properties of steel materials by affecting the state of the microstructure. Therefore, on the basis of commercialized 22MnB5 steel, exploring the influence of micro-alloy element addition and process parameters (austenitizing temperature and holding time) on microstructure and mechanical properties, which is an important theoretical guidance and reference value for the research and development of ultra-high strength hot stamping steel (UHSHSS).

In recent years, the process parameters (such as austenitizing temperature, cooling rate, pressure magnitude, holding time and die exit temperature) in the hot forming process, which directly affect the important factors of its mechanical properties, have been studied intensively and extensively [8,9,10,11,12,13]. Quan et al. [14] investigated in depth the effect of the quenching time and the temperature on the phase-transformation characteristics and mechanical property of ultra-high-strength steel. Jiang et al. [15] closely examined the microstructure and mechanical properties of 22MnB5 steel during hot stamping, and found that the microstructure was uniformly distributed martensite when the austenitization temperature was 930 °C and the holding time was 4.5 min. Mu et al. [16] compared and analyzed the mechanical properties of 30MnB5 and 22MnB5, and the mechanical properties of the hot-formed steel after quenching were the best when the austenitizing temperature was kept at 900 °C and the holding time was 3–5 min. Kong et al. [17] pointed out that the cooling rate has a significant effect on the hardness of 22MnB5 steel after quenching. Zhou et al. [18] concluded that the cooling rate during quenching should exceed 40 °C/s in order to obtain higher strength 22MnB5 hot forming steel. Pedraza et al. [9] believed that a slow heating rate favored the nucleation and growth of austenite, while a fast cooling rate favored the transformation from austenite to martensite. Although many scholars have studied the growth behavior of the austenite grains of 22MnB5 in the hot stamping process, there are few reports on the selection of the hot stamping parameters, microstructure evolution, austenite grain distribution and mechanical properties of UHSHSS after alloying with Nb and V elements. Generally, during the solidification of steel, Nb, V, and other microalloying elements form second-phase particles during the solidification and cooling of steel, which have the effect of pinning grain boundaries and dispersion strengthening [19,20,21,22,23,24]. Liang et al. [25] found that the addition of Nb element can promote the refinement of austenite grains, which has a significant advantage in improving the tensile strength and yield strength of UHSHSS. Lin et al. [26] found that with increasing Nb content in 22MnB5 steel, austenite grains decreased and hardened laths in martensite narrowed. However, the existing austenitization process may not meet the production needs, due to the influence of microalloying on the austenitization temperature, grain size, microstructure and mechanical properties of the steel. Therefore, clarifying the microstructure evolution law during the austenitization process of UHSHSS steel after Nb and V microalloying, which is of practical significance for exploring the synergistic relationship between strength and plasticity, determining the appropriate process and improving comprehensive mechanical properties.

In this study, the effects of austenitizing temperature and holding time on microstructure evolution, grain size and mechanical properties of Nb and V microalloyed UHSHSS have been investigated using a combination of theoretical calculations and experiments. In an attempt to clarify the synergistic control relationship between hot stamping process with microstructure evolution, austenite grain size and mechanical properties, obtaining the suitable austenitizing process parameters for 22MnB5NbV steel. Finally, on the basis of the determined optimal hot forming process, the effects of Nb and V microalloying on the microstructure and properties of hot forming steel are compared and analyzed.

## 2. Materials and Experimental Methods

The chemical compositions of 22MnB5 and 22MnB5NbV used in this study are collected from a commercial steel plant, and the chemical compositions are shown in Table 1. Except Nb and V elements, other components are similar. In order to study the influence of Nb, V and other microalloying elements on the phase transitions of 22MnB5, the CCT curve and phase transitions process of 22MnB5NbV under the existing hot stamping parameters have been calculated by JMatPro thermodynamic software at first. For the existing hot forming process of 22MnB5 steel, the cooling rate during hot stamping is generally greater than 30 °C/s [3,27,28]. The cooling rate of 40 °C/s has been adopted in this study to ensure that the martensitic structure can be obtained as much as possible after quenching. In addition, the holding time is selected as 5 min. The austenitizing temperature has been set at 780 °C~930 °C with an interval of 30 °C.

**Figure 1:**
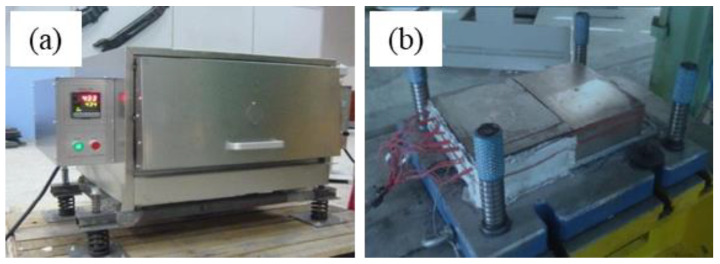
Quenching and heat treatment equipment: (**a**) heating furnace; (**b**) quenching mold.

The dimensions of the quenched steel plates are 250 mm (length) × 250 mm (width) × 1.5 mm (thickness). All quenching and heat treatment tests are performed using a heating furnace and quenching mold. Firstly, the heating furnace is heated up to the set austenitizing temperature and holding for the set time. Then, the steel sample after holding is removed and quickly put into a special mold for quenching treatment. The rapid circulation of cooling water in the quenching mold takes away a large amount of heat from the steel plate. The cooling rate is regulated by adjusting the flow rate of the cooling water. The heating furnace and quenching mold are shown in Figure 1. It is worth noting that during the transfer from the heating furnace to the quenching mold, the temperature of the steel plate decreases to varying degrees. In order to acquire the temperature drop behavior during this process, a handheld infrared temperature gun is used to measure the temperature of steel plate. Each measurement is taken at 1 s intervals until the steel plate is placed in the quenching mold.

**Figure 2:**
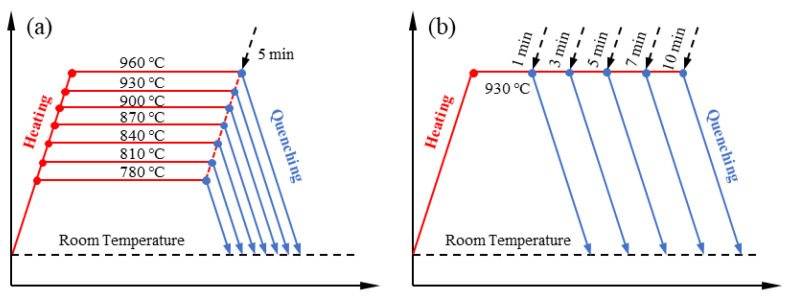
Quenching and heat treatment process: (**a**) different austenitizing temperatures; (**b**) different holding time.

The quenching and heat treatment processes of different austenitizing temperatures and holding times are shown in Figure 2. In the study of the effect of austenitizing temperature (as shown in Figure 2a), in order to fully consider the difference between the experiment and the calculation, the austenitizing temperature has been set at 780 °C~960 °C with an interval of 30 °C, the holding time is 5 min, and the cooling rate is 40 °C/s. In the quenching experiments of different holding time (as shown in Figure 2b), there are five different holding times, and experiments are performed in the range of 1–10 min; the austenitizing temperature is 930 °C, and the cooling rate is 40 °C/s.

After quenching, each steel plate is cut into 10 mm × 10 mm × 1.5 mm microscopic specimens and three standard tensile specimens. After removing oil stains, the microscopic specimens are inlaid with a mosaic machine, and the specimens are ground with sandpaper 120#, 400#, 800#, 1200#, 2000#, and then polishing with a 3.5 μm diamond abrasive agent. Then, polished specimens are etched by 4% (volume fraction) Nital solution for a duration of about 10–15 s. When the surface of the specimens became gray, they are immediately washed with water and then cleaned with alcohol. After being dried by hot air, the required metallographic specimens are obtained. The microstructures of 22MnB5NbV after phase transition can be observed by this method. After that, the specimens are re-ground and polished, and put into the aqueous solution of supersaturated picric acid for repeated thermal erosion until the austenite grain boundary could be clearly observed under the metallographic microscope, and the observed grain sizes are statistically analyzed by using the quantitative metallography method [29].

**Figure 3:**
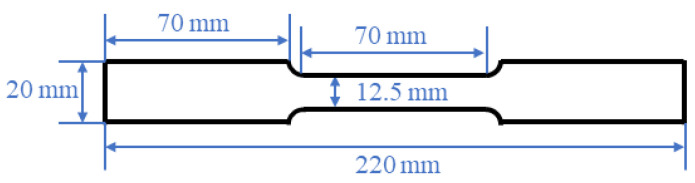
Standard sample size of A_50_.

The tensile specimens are processed according to the A_50_ standard specimen (GB/T228-2002, national standard, China, Beijing) with the dimensions shown in Figure 3. Tensile tests are performed on a SANS CMT5305 tensile test machine at a rate of 3 mm/min at room temperature. In order to avoid the influence of experimental errors on the accuracy of data, the average value of each sample is taken as the final test result after three tests.

Finally, the microstructure and properties of 22MnB5 and 22MnB5NbV have been compared by the same experimental method under the condition of optimal austenitizing parameters, so as to clarify the influence of Nb and V microalloying elements on the properties of UHSHSS.

## 3. Results and Discussion

### 3.1. Phase Transition Calculations of Nb-V-Alloyed UHSHSS under Different Hot Stamping Conditions

In order to determine the influence of existing process parameters on the phase transition of 22MnB5NbV, JMatPro thermodynamic software has been used to calculate the CCT curves of 22MnB5NbV and 22MnB5 steel. Then the phase transitions of 22MnB5NbV at different austenitizing temperatures are calculated. Furthermore, whether the existing process parameters are suitable for the hot stamping process of 22MnB5NbV is analyzed from the thermodynamic point of view.

#### 3.1.1. Continuous Phase Transition at Different Cooling Rates

**Figure 4:**
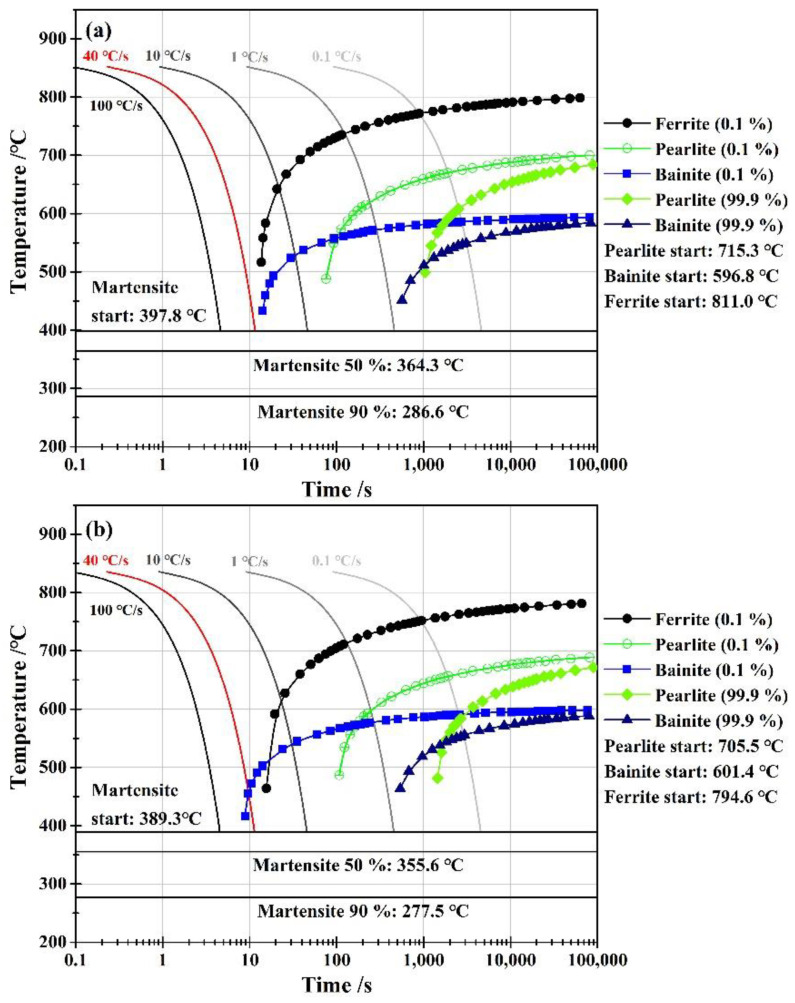
CCT curves of 22MnB5NbV and 22MnB5 steel: (**a**) 22MnB5NbV; (**b**) 22MnB5.

Figure 4 shows the CCT curves of 22MnB5NbV and 22MnB5 steel calculated by JMatPro thermodynamic software. Under the condition of equilibrium phase transition, the austenite decomposition temperatures of the two high-strength steels are 811.0 °C and 794.6 °C, respectively. As for the 22MnB5NbV and 22MnB5 steel, the quenched microstructures contain ferrite, pearlite, bainite and martensite when the cooling rate is 1 °C/s. With the increase of the cooling rate, the pearlite in the microstructures disappears when the cooling rate is 10 °C/s. However, when the cooling rate of the quenching process reaches 40 °C/s, the microstructure of 22MnB5NbV steel is converted into the full martensite, while a certain content of bainite still exists in 22MnB5 steel. Therefore, 22MnB5NbV steel is quenched at a cooling rate of 40 °C/s, which can ensure the ultra-high strength requirements, while 22MnB5 steel requires a higher cooling rate.

#### 3.1.2. Effect of Austenitizing Temperature on Phase Transition

**Figure 5:**
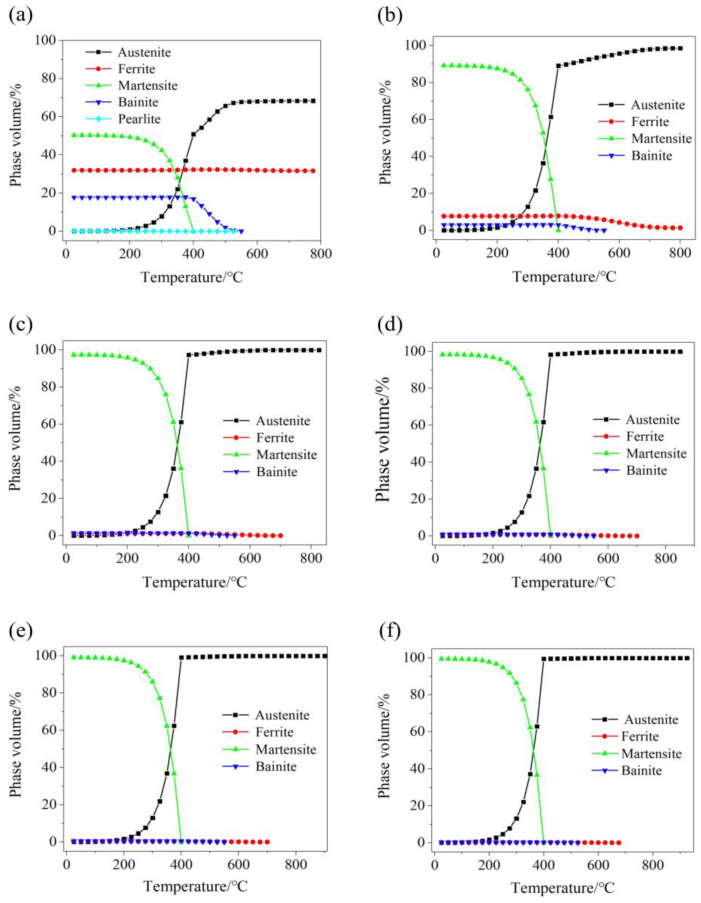
Thermodynamic calculations of the phase transitions of 22MnB5NbV at different austenitizing temperatures: (**a**) 780 °C; (**b**) 810 °C; (**c**) 840 °C; (**d**) 870 °C; (**e**) 900 °C; (**f**) 930 °C.

Figure 5 shows the thermodynamic calculations of phase transition of 22MnB5NbV at different austenitizing temperatures. During the calculations, the holding time is 5 min and the cooling rate is at 40 °C/s. When the austenitizing temperature is at 780 °C, the quenched microstructures contain ferrite and a small amount of pearlite, which are 31.9% and ~0.1%, respectively. At this time, the martensite content is only 50.3%, and the transformation from microstructures to whole martensite is not realized, indicating that the complete austenization of 22MnB5NbV cannot be achieved at 780 °C. When the austenitizing temperature is at 810 °C, the ferrite content is reduced to 7.7%, and the pearlite disappeared, and the martensite content was 89.2%, indicating that at 810 °C, all the pearlite and most of the ferrite had completed the transformation to austenite, and then converted to martensite in the quenching process. The microstructures after quenching still contain ferrite, indicating that the austenitizing temperature at 810 °C cannot make 22MnB5NbV fully austenitized. When the austenitizing temperature is at 840 °C, the ferrite content is 1.2%, and the martensite content is 97.4%, and the bainite content is 1.3%, indicating that 22MnB5NbV basically achieves martensitization after quenching at this temperature. When the austenitizing temperatures are 870 °C, 900 °C and 930 °C, the ferrite contents are 0.7%, 0.4% and 0.2%, and the martensite contents are 98.4%, 99.1% and 99.5%, respectively. The ferrite contents are all low, and the martensite contents are all high, indicating that at these temperatures, the 22MnB5NbV basically realizes the whole martensite after quenching, and the martensite content gradually increases with the increase in austenitizing temperature. Therefore, the austenitizing temperature of 22MnB5NbV should be controlled at least above 870 °C theoretically.

### 3.2. Effect of Austenitizing Temperature on the Microstructures and Mechanical Properties of 22MnB5NbV

#### 3.2.1. Microstructures at Different Austenitizing Temperatures

**Figure 6:**
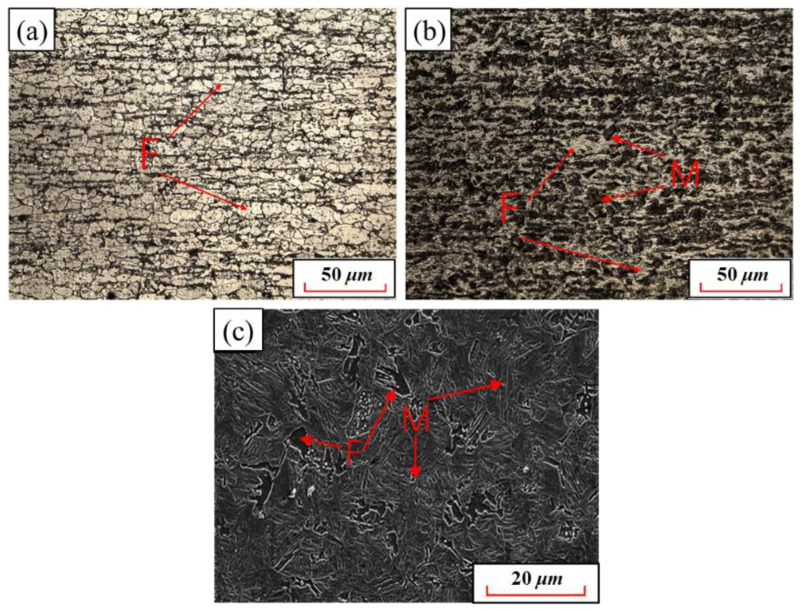
Microstructure of 22MnB5NbV steel after quenching at austenitizing temperature of 780 °C–840 °C: (**a**) 780 °C; (**b**) 810 °C; (**c**) 840 °C.

Figure 6 shows the quenching microstructures of 22MnB5NbV with the austenitizing temperature of 780 °C~840 °C and the holding time of 5 min. It is worth noting that “F” represents ferrite and “M” denotes martensite in this study. When the austenitizing temperature is at 780 °C, the quenched specimen still retains the band characteristics of the original microstructures, and there is fewer ferrite transformed at this time, indicating that 22MnB5NbV is not completely austenitized at this temperature. When the austenitizing temperature is at 810 °C, the ferrite content in the quenched microstructure decreases significantly, indicating that some ferrite dissolves into austenite at this temperature and forms martensite after quenching. When the austenitizing temperature is at 840 °C, the microstructure after quenching is mostly lath martensite, and there are small pieces of ferrite between the martensite. Therefore, when the austenitizing temperature is lower than 840 °C, the complete austenitizing of 22MnB5NbV cannot be completed, which does not reach the required strength of UHSHSS. In order to achieve complete austenization of 22MnB5NbV, it is necessary to further increase the austenitizing temperature.

**Figure 7:**
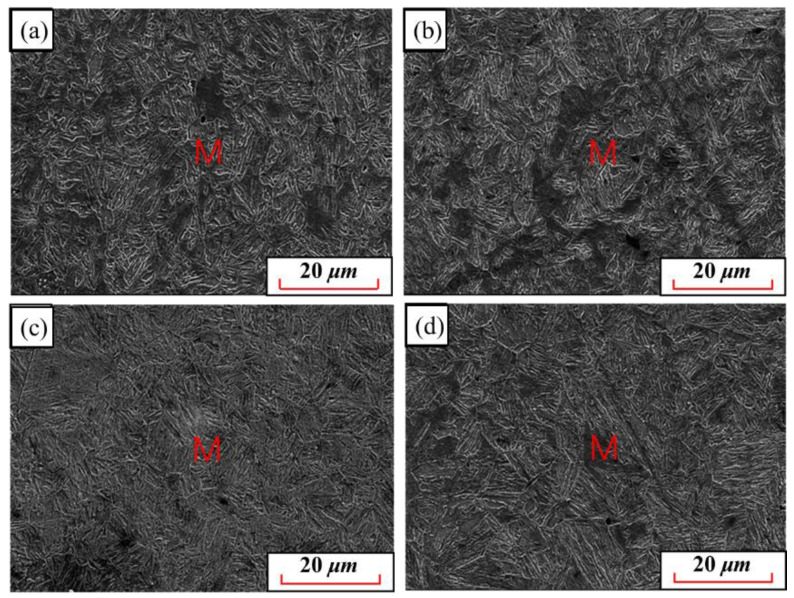
Microstructure of 22MnB5NbV steel after quenching at austenitizing temperature of 870 °C–960 °C: (**a**) 870 °C; (**b**) 900 °C; (**c**) 930 °C (**d**) 960 °C.

Figure 7 shows the quenching microstructures of 22MnB5NbV at 870 °C~960 °C. At this time, the microstructures of 22MnB5NbV after quenching are all lath martensite, and the ferrite has disappeared, indicating that 22MnB5NbV achieves complete austenization at these temperatures. In other words, pearlite and ferrite disappear and transform into austenite, and then transform into whole martensite after quenching. The comparative analysis shows that the martensite lath is smaller when the austenitizing temperature is at 870 °C, and the martensite lath is coarser when the austenitizing temperature is at 960 °C, indicating that the length of martensite lath increases with the increase in temperature. The strength and elongation of UHSHSS decrease with the coarsening of martensitic lath, which is detrimental to the development of the comprehensive mechanical properties of 22Mnb5NbV steel. Therefore, the austenitizing temperature should not be too high.

#### 3.2.2. Average Grain Sizes of Austenite at Different Temperatures

**Figure 8:**
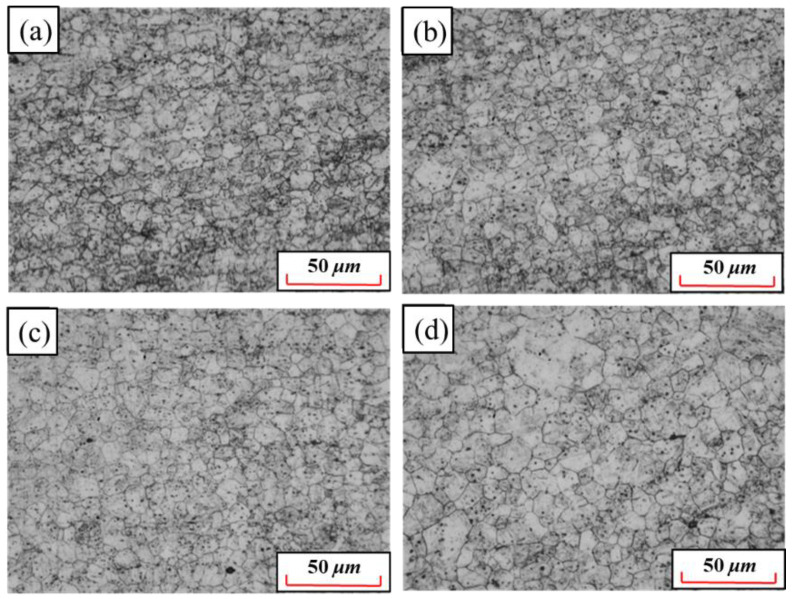
Grain distributions at different austenitizing temperatures: (**a**) 870 °C; (**b**) 900 °C; (**c**) 930 °C (**d**) 960 °C.

**Figure 9:**
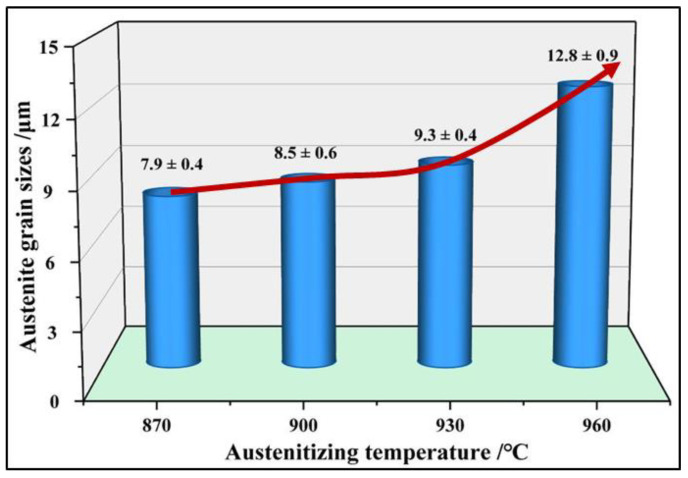
The variation in austenite grain sizes at different austenitizing temperatures.

The present study shows that the formation of martensite is dependent on the grain size of austenite [30]. Moreover, the austenite grain size has an important influence on the microstructure and mechanical properties [31]. Therefore, it is necessary to pay attention to the grain size changes in the austenitizing process of hot formed steel. The grain sizes of 22MnB5NbV after quenching are observed at 870 °C~960 °C, as shown in Figure 8. The grain size increases with the increase in austenitizing temperature. At 870 °C, 900 °C and 930 °C, austenite grain sizes are small and uniformly distributed. While at 960 °C, the austenite grains grow significantly and the grain size distribution is more uneven. From 870 °C to 930 °C, the average grain size increases gently from 7.9 μm to 9.3 μm. When the temperature is at 960 °C, the average size increases obviously, which is negative for the strength and plasticity optimization of 22MnB5NbV, as shown the Figure 9. Therefore, the austenitizing temperature of 22MnB5NbV should be controlled within 930 °C.

#### 3.2.3. Strengths and Elongations at Different Temperatures

**Figure 10:**
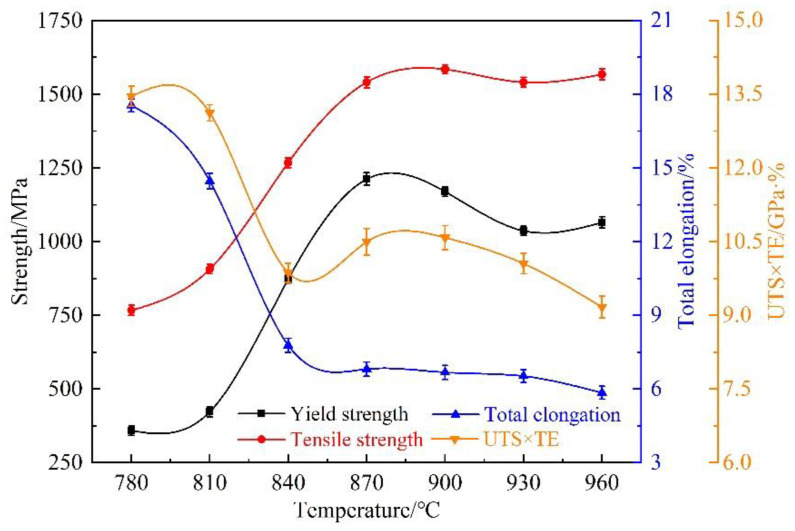
The strength, elongation and UTS × TE variations at different austenitizing temperatures.

Figure 10 shows the variations in the strengths and elongations of 22MnB5NbV at different austenitizing temperatures. When the austenitizing temperature is lower than 870 °C, the tensile strength and yield strength increase with the increase in austenitizing temperature. The reason is that the austenitizing degree of 22MnB5NbV increases with the increase of the temperature, and then the martensite content increases after quenching. The tensile and yield strengths of 22MnB5NbV are basically stable at a high level when the austenitizing temperature exceeds 870 °C. This is due to the transformation of the steel microstructures into austenite during the heating process, and then from austenite to whole martensite in the quenching process. Although 22MnB5NbV will be fully austenitized when the temperature is above 870 °C, its strength will decrease slightly due to the austenite grain growth with the increase in the temperature. The maximum tensile strength reaches 1584.0 MPa at 900 °C.

The elongation of 22MnB5NbV decreases with the increase in the austenitizing temperature. The highest elongation is 17.6% at 780 °C, which is nearly 10.0% lower than the original steel’s elongation at 27.4%. This is due to the transformation of some pearlite and ferrite into austenite in the austenitizing process, and then the transformation of austenite into martensite during quenching. The results show that the elongation decreases significantly in the range of 780 °C to 840 °C, which is due to the transformation of more pearlite and ferrite into austenite with the increase in temperature in the austenitizing temperature range. When the austenitizing temperature is higher than 870 °C, 22MnB5NbV is fully austenitized and transformed into martensite during quenching. At this time, the elongation decreases slowly with the increase in the temperature. When the austenitizing temperatures are at 870 °C, 900 °C, 930 °C and 960 °C, the elongations are 6.8%, 6.7%, 6.5% and 5.9%, and the strength-ductility product are 10.5 GPa·%, 10.6 GPa·%, 10.1 GPa·% and 9.2 GPa·%, respectively. Therefore, in order to ensure the high strength-ductility product of 22MnB5NbV, its austenitizing temperature should be controlled between 870 °C and 930 °C.

**Figure 11:**
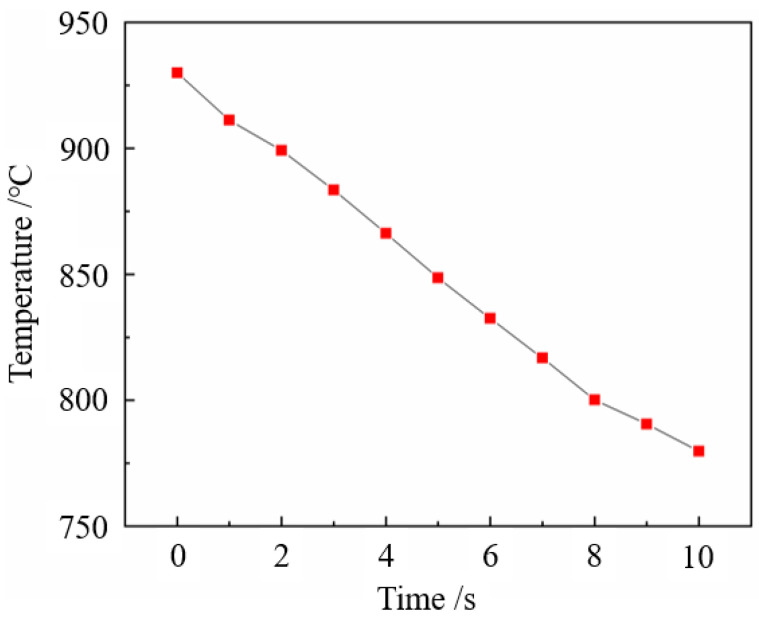
Temperature variation of 22MnB5NbV at air cooling rate from 930 °C.

The microstructures and mechanical properties of 22MnB5NbV are relatively stable in the temperature range of 870 °C to 930 °C. The austenite grain distributions are uniform, and the tensile strength reaches the maximum at 900 °C. Figure 11 shows the temperature variation of 22MnB5NbV steel under air cooling conditions during the actual hot forming process. Apparently, the transfer time of 22MnB5NbV from heating furnace to the die is about 6–7 s, and the temperature drop is about 100 to 120 °C in the actual hot stamping process. According to the previous calculation results, the critical ferrite transition temperature of 22MnB5NbV steel under the condition of equilibrium phase transition is 811 °C. In production practice of the 22MnB5NbV steel, the temperature reduction during the transfer to the mold must be taken into account to avoid the degradation of properties due to insufficient austenitizing process. On the basis of considering the effect of austenitizing temperature on microstructure and properties, combined with the actual hot forming process of 22MnB5NbV steel, the optimal austenitizing temperature should be controlled at 930 °C.

### 3.3. Effect of Holding Time on the Microstructures and Mechanical Properties of 22MnB5NbV

#### 3.3.1. Microstructures after Quenching under Different Holding Time

**Figure 12:**
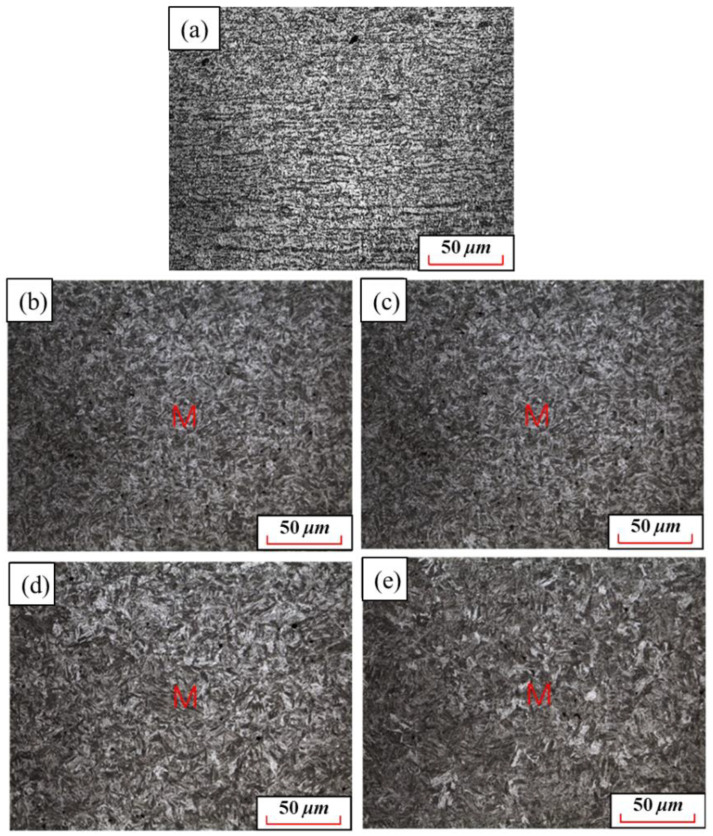
Microstructures of 22MnB5NbV steel at different holding time: (**a**) 1 min; (**b**) 3 min; (**c**) 5 min; (**d**) 7 min; (**e**) 10 min.

Figure 12 shows the quenched metallographic microstructures of 22MnB5NbV at 930 °C with different holding times. When the holding time is 1 min, there are still banded ferrite and pearlite in the specimen, indicating that 22MnB5NbV is not fully austenitized. After quenching, the tensile strength and yield strength of the specimen are 568.5 MPa and 483.8 MPa, which are not significantly improved compared with the original specimen before quenching. The elongation after fracture is 22.6%, which remained at a high level. It shows that the holding time for 1 min cannot make 22MnB5NbV fully austenitized. Therefore, in order to obtain the whole martensite microstructures after quenching, the holding time should be further extended. When the holding time are 3 min, 5 min, 7 min and 10 min, the quenched microstructures are martensite. At this time, all ferrite and pearlite are transformed into austenite, and then the austenite transformed into martensite, which is a powerful guarantee for realizing the ultra-high strength of22MnB5NbV after quenching. With the increase of holding time, martensite becomes coarse gradually, which is not conducive to the increase of its strength-ductility product. Therefore, in order to ensure that 22MnB5NbV with high strength and elongation, the holding time should not be too long.

#### 3.3.2. Original Austenite Grain Size at Different Holding Times

**Figure 13:**
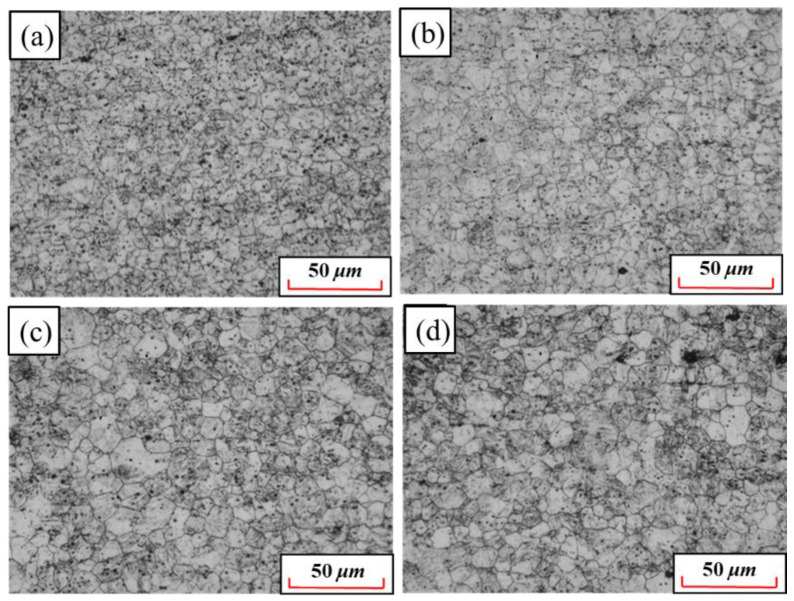
Grain distributions at different holding time: (**a**) 3 min; (**b**) 5 min; (**c**) 7 min; (**d**) 10 min.

**Figure 14:**
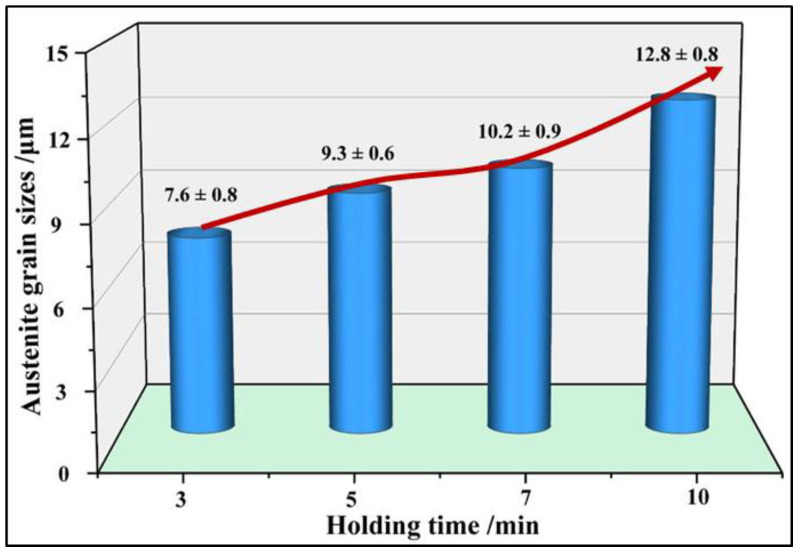
The variation of austenite grain size with different holding time.

Figure 13 and Figure 14 show the grain distributions after quenching at different holding times. The austenite grain size increases with the increase in holding time. When the holding time is 3 min, the austenite grains are small and uniform, and the average grain size is 7.6 μm. When the holding time is 5 min, the grains grow slightly and the distributions are still uniform. When the holding time is 7 min, some grains begin to grow abnormally and the grain distributions begin to become uneven. When the holding time is 10 min, the grain boundary becomes fuzzy. At this time, the average grain size is 12.8 μm and the maximum grain size is about 30 to 40 μm. The increase in austenite grain inhomogeneity is not beneficial for the promotion of the strength and plasticity of 22MnB5NbV after quenching. The microstructures of the steel can be fully austenitized after holding for 3 min. If other conditions permit, holding time of 3 min is more beneficial to the strength and plasticity of 22MnB5NbV.

#### 3.3.3. Strengths and Elongations under Different Holding Time

**Figure 15:**
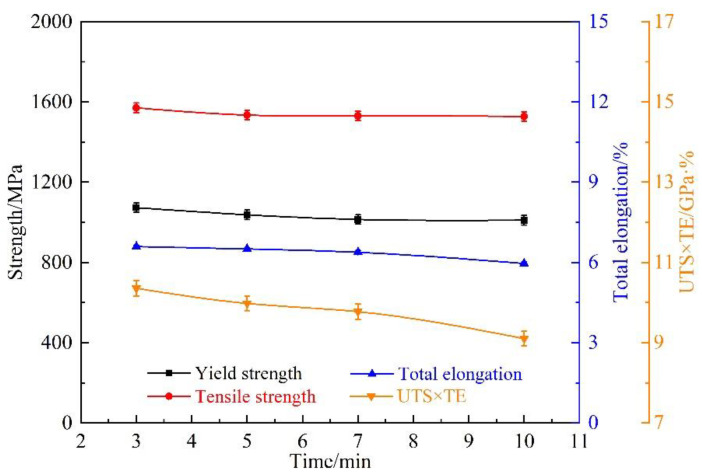
The strength, elongation and UTS × TE variations with different holding time.

Figure 15 shows the variations of strengths and elongations under different holding times. The strength of 22MnB5NbV after quenching decreases slightly with the prolongation of the holding time, which is due to the austenite grain growth. When the austenitizing time is 3 min, the tensile strength and yield strength are 1570.8 MPa and 1076.8 MPa, respectively. When the austenitizing time is 10 min, the tensile strength and yield strength are 1527.1 MPa and 1014.6 MPa, respectively. This result indicates that the prolonged holding time leads to a decrease in mechanical properties. Therefore, it is an effective method to elevate the strength of 22MnB5NbV by shortening the holding time as far as possible on the basis of ensuring complete austenization.

The elongations of 22MnB5NbV decrease with the increase of holding time. When the holding times are 3 min and 5 min, the elongations are 6.6% and 6.4%, respectively. The elongation is relatively close, indicating that the elongation of 22MnB5NbV does not decrease too much when the holding time increases from 3 min to 5 min, which is due to the relatively slow growth of austenite grains. When the holding time is 10 min, the decrease is relatively large, and the elongation is reduced to 6.0%, which is due to the abnormal growth of grains caused by too long holding time. When the holding times are 3 min, 5 min, 7 min and 10 min, the strength-ductility products are 10.4 GPa·%, 10.0 GPa·%, 9.8 GPa·% and 9.1 GPa·%, respectively. When the holding time is 3 min, the strength-ductility product reaches the maximum. By analyzing the evolution processes of microstructures, grain sizes and mechanical properties of 22MnB5NbV, the holding time is finally determined to be 3 min.

### 3.4. Effects of Nb and V Alloying on the Strength and Plasticity of UHSHSS

**Figure 16:**
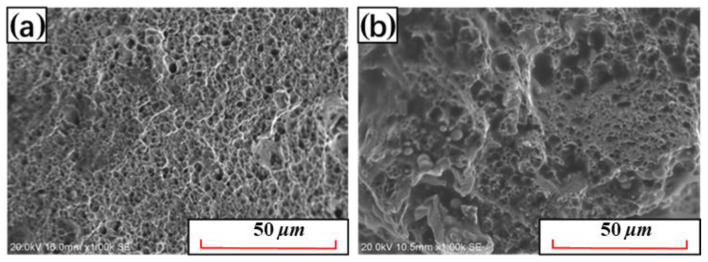
Comparison of fracture surfaces between 22MnB5NbV and 22MnB5 steel: (**a**) 22MnB5NbV, (**b**) 22MnB5.

In order to explore the influence of Nb and V alloying on mechanical properties, the comprehensive properties parameters of 22MnB5NbV and 22MnB5 steel after quenching are listed in Table 2. Since the strength mainly depends on the carbon content in the martensite, the improvement in tensile strength and yield strength is not significant. When carbides are formed in the steel, the carbon content in the martensite decreases, and the strength of the steel even has a certain extent decrease [32]. However, compared with ordinary 22MnB5 steel, the strength-plastic product of 22MnB5NbV is significantly elevated, especially the elongation rate has increased by nearly 0.6%. The fracture surfaces of 22MnB5NbV and 22MnB5 steel are shown in Figure 16. Apparently, the fracture surfaces of 22MnB5NbV and 22MnB5 steel are mainly covered by dimples with different depths, and have the typical fracture morphology of dimple-plastic fracture. Compared with 22MnB steel, it can be clearly found that the dimples at the fracture of 22MnB5NbV are smaller, denser and more uniform.

**Figure 17:**
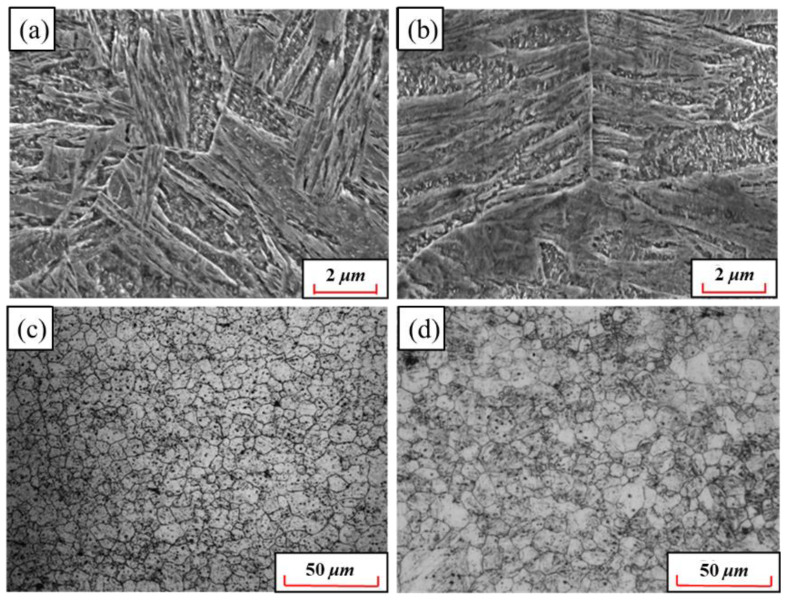
Comparison of microstructures and grains between 22MnB5NbV and 22MnB5 steel: (**a**) microstructure of 22MnB5NbV; (**b**) microstructure of 22MnB5; (**c**) grain of 22MnB5NbV; (**d**) grain of 22MnB5.

The difference in fracture surfaces of 22MnB5NbV and 22MnB5 steel mainly depend on the microstructure state. Figure 17 shows the microstructures and grain sizes comparison between 22MnB5NbV and 22MnB5 after quenching. The martensitic lath of 22MnB5NbV is mainly of fine and short shape, and its length is obviously smaller than that of 22MnB5 steel. The average grain size of 22MnB5NbV is 7.6 μm and that of 22MnB5 is 11.7 μm. Generally, a finer grain size will show better plasticity in the same microstructure.

**Figure 18:**
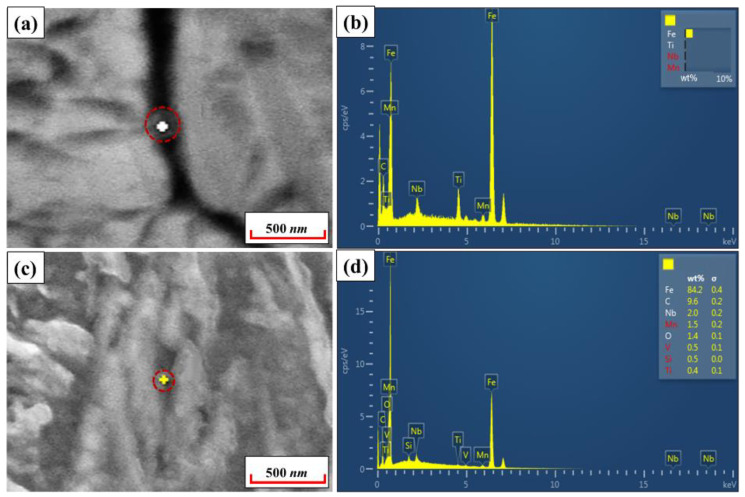
EDS analysis of grain boundaries and intragranular precipitates in 22MnB5NbV steel: (**a**) and (**b**) are for the grain boundaries; (**c**) and (**d**) are for the grain intragranular.

In order to explore the essential reasons for the difference in the grain sizes of 22MnB5NbV and 22MnB5 steel, the precipitates at the grain boundaries and within the grains have been detected and analyzed by scanning electron microscopy, the result is shown in Figure 18. The detection results showed that a certain number of precipitates appeared in the grain boundaries and in the grains, of which the size of the precipitates is basically in the range of 100–200 nm. Meanwhile, the type of precipitation is mainly composed of (Nb, Ti, V) carbides. Current research generally agrees that micron-sized precipitates in steel can lead to compromised benefits of alloying and even cause degradation of mechanical properties [29,33,34]. However, diffusely distributed nanoscale nitrides or carbonitrides can effectively pin down grain boundaries, hindering grain growth and significantly improving the strength and toughness of the material [33,35,36]. Therefore, the addition of Nb and V elements can significantly inhibit the growth of grains through the strengthening effect of the precipitation phase, and the fine grains are beneficial to the further increase the elongation and the strong-plastic product of UHSHSS.

## 4. Conclusions

In this research, the 22MnB5NbV steel microalloyed with Nb and V elements has been used as the research material. The effects of Nb and V alloying elements on the microstructure and properties of hot stamping steel are studied. The suitable parameters of the hot stamping process for 22MnB5NbV steel microalloyed with Nb and V elements are formulated. The following conclusions can be drawn:

(1) The microstructures and grain sizes of 22MnB5NbV gradually become coarse with the increase of austenitizing temperature and holding time. When the austenitizing temperature is at 930 °C and the holding time is 3 min, the average grain size, the tensile strength, elongation and strength-ductility product at room temperature are 7.7 μm, 1570.8 MPa, 6.6% and 10.4 GPa·%, respectively.
(2) The 22MnB5NbV steel can be completely austenitized, while the austenitizing temperature ≥870 °C and the holding time ≥3 min. Based on the comparison of microstructure, grain size and comprehensive properties, combined with production practice, the optimum austenitizing temperature and holding time is 930 °C and 3 min.
(3) The addition of Nb and V elements promotes the formation of nanoscale precipitates on and within the grain boundaries. Under the optimal austenitizing process conditions, the elongation and strength-plastic product of the microalloyed 22MnB5NbV steel increased by ~0.6% and ~1.2 GPa·% compared with the 22MnB5 steel.

## Figures and Tables

**Table 1:** Composition of 22MnB5NbV and 22MnB5 (wt.%).

Steel	C	Si	Mn	P	S	Al	Cr	B	Ti	Nb	V	Fe
22MnB5NbV	0.24	0.23	1.24	0.013	0.002	0.04	0.16	0.003	0.040	0.04	0.042	balance
22MnB5	0.23	0.34	1.47	0.010	0.003	0.04	0.18	0.002	0.040	/	/	balance

**Table 2:** Comparison of mechanical properties between 22MnB5NbV and 22MnB5 steel.

Steel	Tensile Strength/MPa	Yield Strength/MPa	Elongation/%	Strength-Ductility Product/GPa·%
22MnB5NbV	1570.8 ± 10.3	1076.8 ± 8.3	6.6 ± 0.1	10.4 ± 0.2
22MnB5	1538.7 ± 11.1	1031.0 ± 9.6	6.0 ± 0.1	9.2 ± 0.1

## Data Availability

All data generated or analyzed during this study are included in this published article. Experimental data used in this study are available from the corresponding author upon reasonable request.
